# Investigating biomarkers for personality alterations in temporal lobe epilepsy patients: based on peripheral inflammatory indices, electroencephalography, and neuroimaging

**DOI:** 10.3389/fpsyt.2025.1622726

**Published:** 2025-07-28

**Authors:** Jia Wang, Fuchi Zhang, Yunshan Zhou, Xiulin Zhang, Jianyang Xu, Shouyong Wang, Chengbing Huang, Taipeng Sun, Hugen Xu, Xiangsong Shi

**Affiliations:** ^1^ Department of Psychiatry, Huai’an No.3 People’s Hospital, Huai’an, China; ^2^ Department of Neurology, Huai’an No.3 People’s Hospital, Huai’an, China

**Keywords:** temporal lobe epilepsy, personality alterations, inflammatory biomarkers, magnetic resonance imaging, video electroencephalography

## Abstract

**Background:**

The investigation of personality alterations in temporal lobe epilepsy (TLE) constitutes a complex and demanding field of research. These alterations may be intricately linked to neuroinflammation, imaging changes, and electrophysiological irregularities.

**Objective:**

This study aims to explore the potential value of the peripheral inflammatory indices, video electroencephalogram (VEEG), hippocampal magnetic resonance imaging (MRI) as biomarkers for personality changes in patients with TLE.

**Methods:**

A total of 110 individuals with TLE were categorized into two groups: 55 patients exhibiting personality alterations and 55 patients without personality abnormalities. A supplementary cohort of 150 healthy individuals was enlisted as a control group. Demographic information, clinical attributes, inflammatory biomarkers, hippocampus MRI, and video EEG data were gathered and subjected to statistical analysis utilizing SPSS software.

**Results:**

In comparison to the healthy control group, patients with TLE demonstrated markedly reduced counts of neutrophils, lymphocytes, and platelets, although the monocyte-to-lymphocyte ratio (MLR) was considerably elevated (all *P*<0.05). The cohort exhibiting personality alterations demonstrated an extended disease duration, an elevated incidence of hippocampal sclerosis or atrophy on MRI, and a reduced rate of monotherapy relative to the cohort without personality alterations (*P*<0.05). Binary logistic regression research indicated no significant correlation between personality alterations in patients with TLE and neutrophil-to-lymphocyte ratio (NLR), platelet-to-lymphocyte ratio (PLR), MLR, systemic immune-inflammation index (SII), or pan-immune-inflammation value (PIV).

**Conclusions:**

MLR was markedly elevated in patients with TLE relative to healthy controls. Hippocampal sclerosis or atrophy constituted an independent risk factor for personality alterations in TLE, although monotherapy seemed to serve as a protective factor.

## Introduction

Epilepsy is a prevalent chronic disorder of the central nervous system, with epidemiological studies indicating a frequency of 61.44 per 100,000 years (95% confidence interval [CI] 50.75−74.38), and it may present at any age (1). Temporal lobe epilepsy (TLE) is the predominant form of focal epilepsy in adults, characterized by seizures that originate in the medial temporal lobe (e.g., hippocampus, amygdala, parahippocampal gyrus) or the lateral temporal neocortex. Research indicates that individuals with TLE are at an increased risk for concomitant mental disorders, likely attributable to the limbic system’s role in regulating emotions and behavior (2). Nonetheless, for an extended period, investigations by epileptologists and psychiatrists have predominantly concentrated on psychosis, anxiety disorders, and mood disorders, whereas personality alterations in patients with epilepsy have been comparatively neglected.

Individuals with epilepsy who comorbid personality alterations generally show lower medication compliance and may face misunderstanding and discrimination in society. These problems not only impact their social life but also undermine familial unity, imposing additional psychological responsibilities and stress on family members. Especially in patients with TLE, comorbidity with personality changes is common and may even be an indicator of adverse outcomes (3, 4). Consequently, the prompt recognition and intervention of personality alterations are essential for the thorough management of TLE. Nonetheless, most research on personality alterations has relied on psychometric assessment tools, and in clinical practice, we lack definite biological markers. A strong association has been reported between levels of inflammatory markers and personality alterations (5). Considering the significant role of inflammation in the aetiology of epilepsy, affecting neuronal transmission and synaptic plasticity, it is inferred that inflammation may modify emotional and behavioral patterns in people with epilepsy (6, 7). Inflammation-related markers could act as possible biological indicators of personality alterations in TLE. However, there are no published studies that have examined this topic in detail and more research is required.

The neutrophil-to-lymphocyte ratio (NLR), platelet-to-lymphocyte ratio (PLR), monocyte-to-lymphocyte ratio (MLR), systemic immune-inflammation index (SII), and pan-immune-inflammation value (PIV) are emerging indicators of systemic inflammation that are extensively acknowledged. These markers are swiftly and economically acquired in clinical environments and have been extensively utilized in evaluating disease severity and prognosis in disorders like epilepsy. Prior studies have identified a correlation between NLR and the incidence of seizures as well as mood disorders (8). Saccaro et al. (9)have established a link between personality problems and peripheral inflammation. The correlation between these novel inflammatory indicators and personality alterations in TLE is still ambiguous. Moreover, research indicates that atypical hippocampal magnetic resonance imaging (MRI) findings constitute a risk factor for psychiatric disorders in TLE (10), and there have also been investigations employing electroencephalography (EEG) to forecast personality traits (11). Consequently, we examined NLR, PLR, MLR, SII, PIV, video EEG, and hippocampus MRI in 110 patients with TLE to investigate the possible correlation of these markers with alterations in personality.

## Materials and methods

### Study participants

This study encompassed patients diagnosed with TLE at Huai’an NO.3 People’s Hospital from January 2020 to December 2022. Patients were stratified into two distinct groups based on their scores on the Eysenck Personality Questionnaire (EPQ): one group comprised individuals exhibiting significant personality alterations as defined by specific EPQ criteria, and the other group included those without detectable personality abnormalities according to the same criteria. We referred to previous relevant studies (12) to determine a sample size of 55 participants for both the personality change group and the group without personality abnormalities, ensuring sufficient statistical power to detect significant differences in personality traits as assessed by the Eysenck Personality Questionnaire (EPQ). Recruitment for each group was executed consecutively; once the goal sample size for one group was attained, its recruitment ceased, while the other group proceeded until its sample size requirement was fulfilled. The diagnosis of TLE adhered to the criteria established by the International League Against Epilepsy (ILAE) (13), necessitating the fulfillment of at least two criteria, with criterion (1) being obligatory: (1) clinical manifestations indicative of an epileptic focus in the temporal lobe; (2) MRI revealing unilateral or bilateral hippocampal sclerosis or atrophy in the temporal lobe; (3) EEG demonstrating spikes or sharp waves in the temporal lobe. The inclusion criteria were: (1) age >16 years; (2) receiving stable antiseizure medications (ASMs) regimens (i.e., no changes in ASMs type or dosage for at least 4 weeks prior to enrollment); (3) possessing at least a junior high school education and capable of completing neuropsychological assessments; (4) absence of seizure occurrences in the preceding 2 weeks. The exclusion criteria comprised: (1) acute infectious diseases; (2) a history of traumatic brain injury, stroke, or encephalitis; (3) chronic conditions, such as hematological disorders or autoimmune diseases; (4) prior use of antibiotics or immunosuppressants within the preceding month; (5) other somatic illnesses resulting in personality alterations; and (6) a history of additional psychiatric disorders. Furthermore, 150 healthy individuals were incorporated as a control group.

The research received approval from the Ethics Committee of Huai’an NO.3 People’s Hospital (Approval Number: 2019-020) and was executed in alignment with the principles of the Declaration of Helsinki. All participants provided informed consent.

### Methods

Clinical data encompassing sex, age, years of education, disease duration, hippocampus MRI findings, video EEG, antiseizure drugs, and EPQ scores were gathered from patients with TLE. Demographic data, including sex, age, and years of schooling, were obtained solely for the healthy control group.

Routine blood tests were conducted utilizing the fully automated hematological analyzer BC-5390CRP (Mindray Co.) (14). Collected blood parameters comprised white blood cell count, neutrophil count, lymphocyte count, monocyte count, and platelet count. According to these parameters, the following calculations were performed: NLR = neutrophil count/lymphocyte count; PLR = platelet count/lymphocyte count; MLR = monocyte count/lymphocyte count; SII = platelet count × neutrophil count/lymphocyte count; PIV = neutrophil count × monocyte count × platelet count/lymphocyte count.

Video EEG monitoring was performed utilizing the Nihon Kohden 1100K video EEG equipment, with a minimum duration of 4 hours of recording. Data were acquired from 19 electrodes placed on the scalp based on the International 10/20 system: frontal F - Fp 1/2, F 3/4/7/8, centroparietal -C 3/4, P 3/4, temporal T - T 3/4/5/6, and occipital O - O 1/2 (15). In this study, we focused on the electrode pairs F7/8 and T3/4/5/6. Two independent EEG professionals analyzed the EEG reports.

Hippocampal MRI: MRI scans were conducted with a 1.5 T superconducting MRI scanner (uMR560, United Imaging Healthcare). Patients underwent scanning in a supine position with their eyes closed, utilizing a 16-channel head-neck combination coil. The MRI scan sequences and parameters were as follows (1): Sagittal T1-weighted imaging (T1WI) sequence: Field of view (FOV) 240 mm × 230 mm, slice thickness 3.0 mm, slice gap 0.3 mm, repetition time (TR)/echo time (TE): 686 ms/13.2 ms, matrix 240 × 160, bandwidth 120 Hz/pixel, number of averages 1, scan duration 1 min 54 s; (2) Axial T2-weighted imaging (T2WI) sequence: FOV 180 mm × 180 mm, slice thickness 3.0 mm, slice gap 0.3 mm, TR/TE: 5000 ms/80.78 ms, matrix 256 × 180, bandwidth 190 Hz/pixel, number of averages 1.5, scan duration 1 min 46 s; (3) Coronal T2 fluid-attenuated inversion recovery (T2FLAIR) sequence: FOV 230 mm × 200 mm, slice thickness 3.0 mm, slice gap 0.3 mm, TR/TE: 8145 ms/82.6 ms, matrix 256 × 200, bandwidth 150 Hz/pixel, number of averages 1, scan duration 2 min 27 s. The MRI reports were collaboratively examined by two radiologists.

The Eysenck Personality Questionnaire (EPQ) (16) was given by a psychiatrist with standardized training. Personality alterations were evaluated utilizing the EPQ, including four temperamental dimensions: Psychoticism (P), Extraversion (E), Neuroticism (N), and Lie (L). The questionnaire was evaluated with standardized T-scores. A T-score exceeding 70 on the L scale signifies a pronounced inclination towards concealment, resulting in the exclusion of such surveys as invalid. Personality alterations were characterized by T-scores < 38.5 or > 61.5 on any of the P, E, or N scales.

### Statistical analysis

Statistical analyses were conducted with SPSS 26.0 software. Experimental data were presented as means ± standard deviations. The Student’s t-test was utilized for continuous variables that are normally distributed or substantially normally distributed. For continuous variables that do not adhere to a normal distribution, pairwise rank-sum tests were utilized. The chi-square test was employed to assess categorical data. Binary logistic regression was employed for multivariate analysis. A *p*-value of less than 0.05 was deemed statistically significant.

## Results

### Comparison of demographic data among study participants

Comparisons of demographic data among the three groups—patients with TLE having personality alterations, those without personality abnormalities, and the healthy control group—revealed no statistically significant differences in sex, age, or years of schooling (all *P* > 0.05), as reported in **Table 1**.

**Table 1:** Clinical and demographic information of controls and patients with temporal lobe epilepsy (TLE).

Demographic data	TLE with personality alterations (n=55)	TLE without personality abnormalities (n=55)	Control group (n=150)	*X^2^* or *F*	*P*
Gender male	30 (54.5%)	32 (58.2%)	87 (58.0%)	0.218	0.897
Female	25 (45.5%)	23 (41.8%)	63 (42.0%)		
Age, years	42.91 ± 13.27	39.80 ± 12.71	41.24 ± 10.83	0.959	0.385
Years of education	11.20 ± 3.02	10.47 ± 2.57	11.19 ± 2.71	1.496	0.226

### Comparative analysis of clinical features in TLE with and without personality alterations

In comparison to the TLE without personality alterations, the cohort with personality abnormalities demonstrated an extended disease duration and an elevated incidence of hippocampus MRI sclerosis or atrophy, although the rate of monotherapy was markedly reduced. The differences were statistically significant (*P* < 0.05), as stated in **Table 2**.

**Table 2:** Comparison of clinical characteristics in patients with TLE patients with and without personality alterations.

Clinical characteristics	TLE with personality alterations (n=55)	TLE without personality abnormalities (n=55)	*t* or *X^2^*	*P*
Disease duration (years)	18.76 ± 11.87	12.73 ± 11.55	2.704	<0.001
VEEG-left (IED),n (%)	49 (89.1%)	45 (81.8%)	1.170	0.209
VEEG-right (IED),n (%)	36 (65.5%)	35 (63.6%)	0.040	0.500
MRI (hippocampal sclerosis or atrophy), n (%)	26 (47.3%)	10 (18.2%)	10.571	0.002
ASM_Single, n (%)	6 (10.9%)	24 (68.6%)	14.850	<0.001

### Comparison of inflammatory markers among three groups

**Figure 1:**
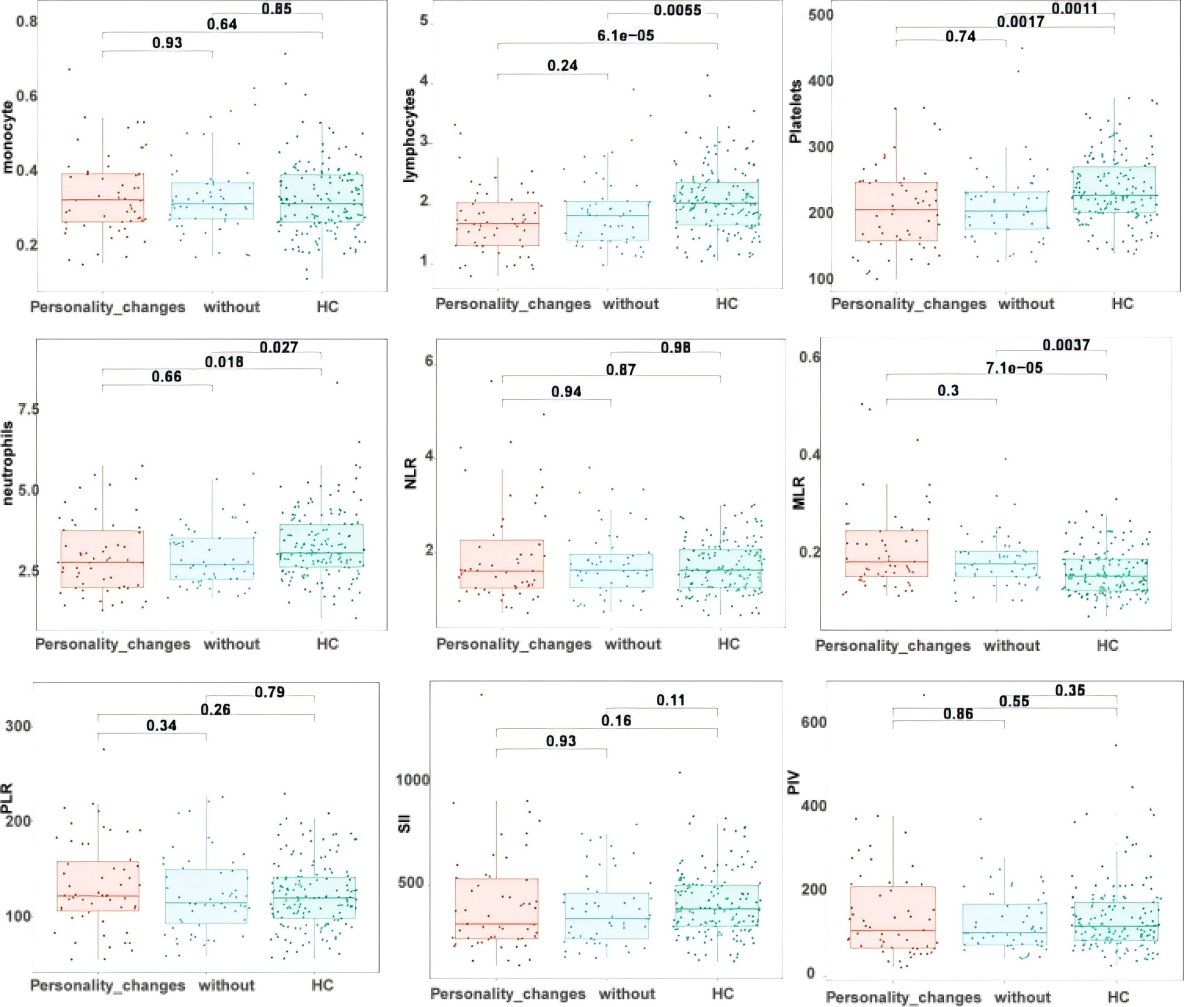
Comparative analysis of the TLE group with the healthy control group.

A study of inflammatory markers among the three groups—patients with TLE having personality alterations, those without personality abnormalities, and the healthy control group—demonstrated substantial differences. In comparison to the healthy control group, individuals with TLE, regardless of personality alterations, demonstrated significantly reduced neutrophil, lymphocyte, and platelet counts, alongside a notably increased MLR (all *P* < 0.05). Nonetheless, no significant changes were observed in monocyte counts, NLR, PLR, SII, and PIV among the three groups (*P* > 0.05), as depicted in **Figure 1**.

### Video EEG monitoring

All patients had video EEG monitoring, revealing unilateral or bilateral temporal lobe spikes and strong wave discharges during the interictal phase. In total, 94 individuals displayed left-sided discharges, 49 of them experienced concomitant personality alterations; 71 patients exhibited right-sided discharges, with 36 presenting personality alterations.

### Hippocampal MRI scanning

All patients received hippocampus MRI scanning, which identified hippocampal atrophy or sclerosis in 36 cases (32.7%), with 20 of these patients exhibiting concomitant personality alterations.

### Binary logistic regression study comparing TLE with and without personality alterations

**Figure 2:**
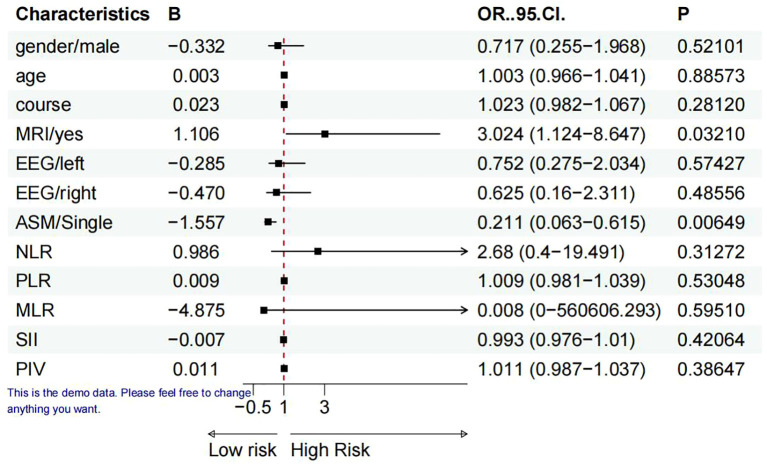
Predictors of TLE accompanied with personality alteration (forest plot). Variables utilized in binary logistic regression analysis: sex, age, disease progression, hippocampal MRI (hippocampal sclerosis or atrophy=yes), EEG (left and right), ASM, NLR, PLR, MLR, SII, and PIV.

A binary logistic regression analysis was performed utilizing sex (men=1, women=2), age, disease duration, hippocampal MRI (hippocampal sclerosis or atrophy=yes), VEEG-left (positive=1), VEEG-right(positive=1), ASM (single=1, combined=2), NLR, PLR, MLR, SII, and PIV as independent variables, while the presence of personality alterations (present=1, absent=2) served as the dependent variable. The key factors revealed in the regression model were hippocampus MRI and ASM, as depicted in **Figure 2**.

## Discussion

Our study demonstrates that patients with TLE display enduring chronic inflammation during interictal intervals. Moreover, chronic inflammation, hippocampal sclerosis or atrophy, and antiseizure drugs may significantly correlate with personality alterations in people with TLE. This implies that these factors may further influence the long-term prognosis of TLE, highlighting the necessity for ongoing monitoring of inflammatory responses, hippocampus MRI, and evaluation of personality alterations in the long-term therapy of TLE.

This investigation revealed that the MLR in patients with TLE was significantly elevated compared to healthy controls, whereas the numbers of neutrophils, lymphocytes, and platelets were notably reduced. Despite the absence of notable variations in NLR, PLR, SII, and PIV relative to the control group, the increased MLR signifies the existence of ongoing chronic inflammation in patients with TLE, even during interictal phases. Prior research has indicated comparable inflammatory responses during acute seizures, and the NLR has been validated as a significant marker for evaluating seizure frequency and severity (14, 17). This implies that NLR exhibits more sensitivity during the acute phase of seizures, while MLR is more closely linked to inflammatory alterations during interictal intervals. Our literature review on PubMed for studies examining inflammatory responses in patients with TLE during interictal periods revealed a limited number of findings employing new inflammatory markers (18). Our findings indicate that increased MLR may function as a possible biomarker for systemic inflammatory responses in TLE. Frigerio et al. (19) noted in animal models of epilepsy that lymphocyte and monocyte activation resulted in elevated IL-1β levels, which disrupted the blood-brain barrier and facilitated lymphocyte migration into brain tissue, a critical pathological mechanism of immune-mediated inflammatory responses. Furthermore, previous research indicates that MLR levels are markedly elevated in individuals with chronic mental conditions, including mood disorders and schizophrenia, in comparison to healthy controls (20, 21). These findings highlight the significance of persistent inflammation in neuropsychiatric disorders. Our findings validate the strong correlation between the development of epilepsy and systemic inflammatory responses. Moreover, they propose that MLR may significantly influence the chronic mechanisms by which inflammation modifies synaptic and neuronal excitability, suggesting that the monitoring and regulation of inflammatory markers such as MLR in the management of TLE could be essential for enhancing patients’ long-term outcomes. Regrettably, we did not identify an association between inflammatory markers, such as MLR, and personality alterations in TLE.

This study revealed that the positive detection rate of hippocampus MRI sclerosis or atrophy in patients with TLE was 32.7%. Among these individuals, those with personality alterations had a considerably elevated detection rate of 72.2%, in contrast to 27.8% in patients without such changes. Numerous studies indicate that in patients with mesial TLE, the prevalence of hippocampal anomalies may attain 50−60% (22–24). The correlation between hippocampal sclerosis and personality alterations in TLE is probably associated with an elevated risk of malfunction in the frontotemporal network. Rivera Bonet et al. (25) identified a negative connection between hippocampal and amygdala volumes and neuroticism in a study including 67 patients with TLE. Moreover, Oskar Zarnowski et al. (26) discerned atypical activation patterns in the limbic region of individuals with personality disorders via resting-state functional MRI investigations. Based on prior publications and our findings, we propose that hippocampus sclerosis or atrophy may function as a biological marker for personality alterations in TLE. In our comparative investigation of interictal epileptiform discharges (IEDs) across the two groups, no significant link was observed for personality alterations, irrespective of left or right temporal lobe discharges. This corresponds with the results of Bragatti et al. (27)in their investigation of IEDs in 78 patients with TLE. Consequently, patients with TLE exhibiting structural abnormalities in the hippocampus may be more prone to personality alterations, indicating that MRI could offer superior predictive value for risks compared to video EEG.

Our research demonstrates that an extended duration of epilepsy correlates with a heightened probability of concurrent personality alterations, but monotherapy acts as a protective factor against personality alterations in TLE. Prior studies have established that, alongside disease duration, an earlier age of onset and increased seizure frequency are risk factors for personality alterations in TLE (28). Additional evidence indicates that antiepileptic medicines (ASM) may influence the psychological and behavioral dimensions of patients with epilepsy, especially levetiracetam and perampanel (29, 30). The negative neuropsychiatric effects associated with ASMs are linked to dosage, titration rate, serum concentration, and drug type. A cross-sectional investigation shown that polytherapy was linked to a greater occurrence of adverse effects in comparison to monotherapy (31). The utilization of numerous antiseizure drugs may elevate the chance of concomitant personality alterations, underscoring the need of favoring monotherapy in the management of TLE.

Binary logistic regression study reveals that hippocampal anatomical abnormalities and antiepileptic drugs serve as independent risk variables for personality alterations in patients with TLE. This discovery directs our attention to the structural alterations in the hippocampus during the therapy of TLE. Additionally, patients exhibiting anatomical abnormalities in the hippocampus and receiving combination antiepileptic medicines should be meticulously watched for the potential emergence of personality alterations.

## Conclusion

This study concluded that MLRs in patients with TLE were markedly elevated compared to those in healthy controls. Additionally, patients with TLE with an extended illness duration, concurrent hippocampal sclerosis or atrophy, and those on polytherapy face an elevated risk of experiencing personality alterations. Hippocampal sclerosis or atrophy acts as an independent risk factor for personality alterations in TLE, whereas monotherapy functions as a protective factor. Our findings will aid in directing the therapy and prognosis evaluation of TLE.

## Limitations

This study has several limitations. First, this study did not account for whether the subjects were patients with drug-resistant epilepsy (DRE) as a confounding factor, which may introduce bias to the analysis of associations between inflammatory markers, neuroimaging features and personality alterations. Second, during the study design phase, we failed to consider the confounding influences of seizure frequency and the particular categorization of antiepileptic drugs. Subsequent study ought to concentrate on identifying a very homogeneous cohort of individuals exhibiting comparable seizure frequencies, while regulating the sort of antiseizure drugs used. Finally, utilizing multimodal MRI techniques focused on the hippocampus and amygdala will provide a more comprehensive exploration of the pathophysiological pathways that contribute to personality alterations during the interictal phase of TLE.

## Data Availability

The raw data supporting the conclusions of this article will be made available by the authors, without undue reservation.
