# Correction: Investigating biomarkers for personality alterations in temporal lobe epilepsy patients: based on peripheral inflammatory indices, electroencephalography, and neuroimaging

**DOI:** 10.3389/fpsyt.2025.1691295

**Published:** 2025-09-10

**Authors:** Jia Wang, Fuchi Zhang, Yunshan Zhou, Xiulin Zhang, Jianyang Xu, Shouyong Wang, Chengbing Huang, Taipeng Sun, Hugen Xu, Xiangsong Shi

**Affiliations:** ^1^ Department of Psychiatry, Huai’an No.3 People’s Hospital, Huai’an, China; ^2^ Department of Neurology, Huai’an No.3 People’s Hospital, Huai’an, China

**Keywords:** temporal lobe epilepsy, personality alterations, inflammatory biomarkers, magnetic resonance imaging, video electroencephalography

The figures, but not the captions, were published in the wrong order. The order has now been corrected.

The original version of this article has been updated.

**Figure 1:**
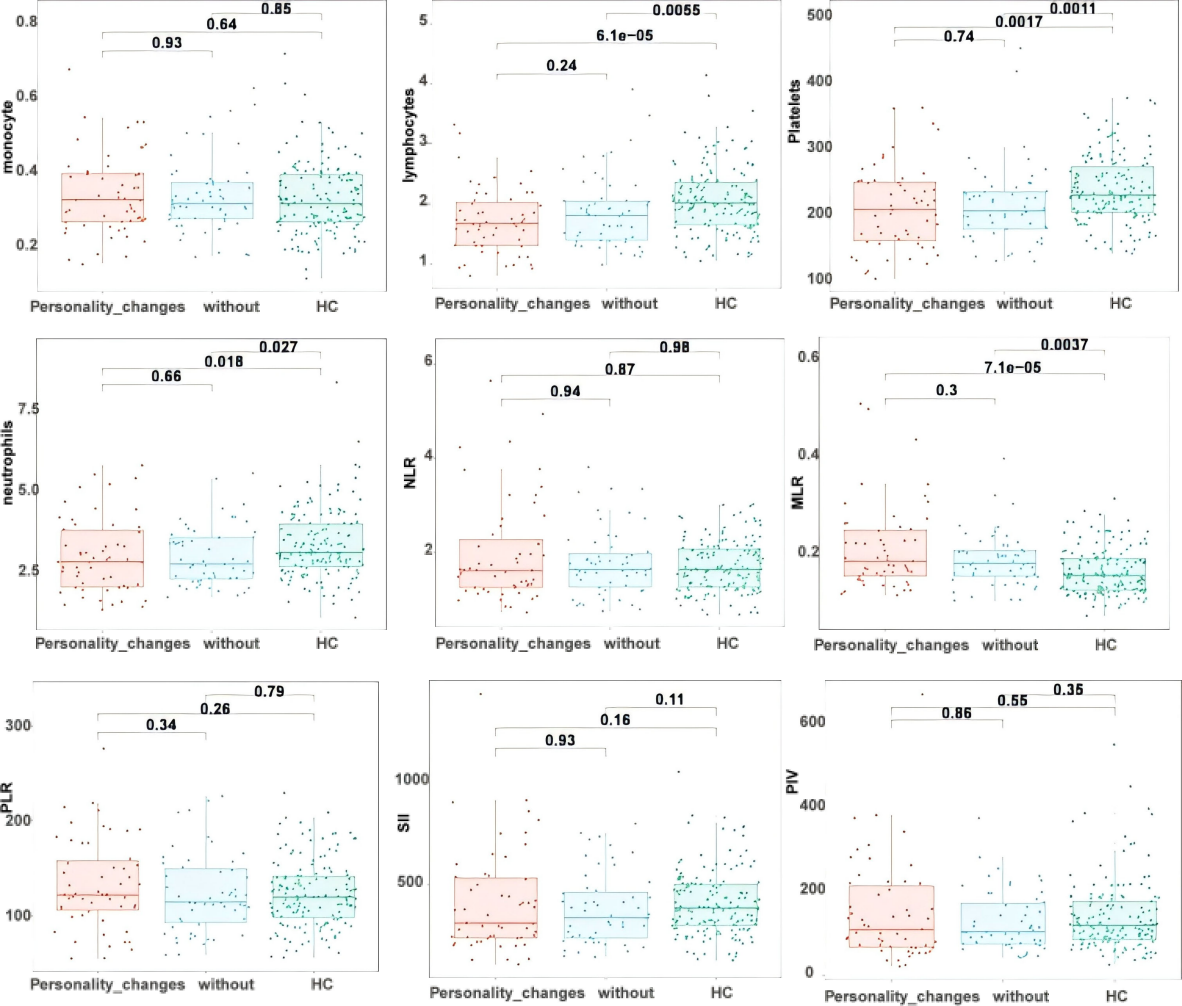
Comparative analysis of the TLE group with the healthy control group.

**Figure 2:**
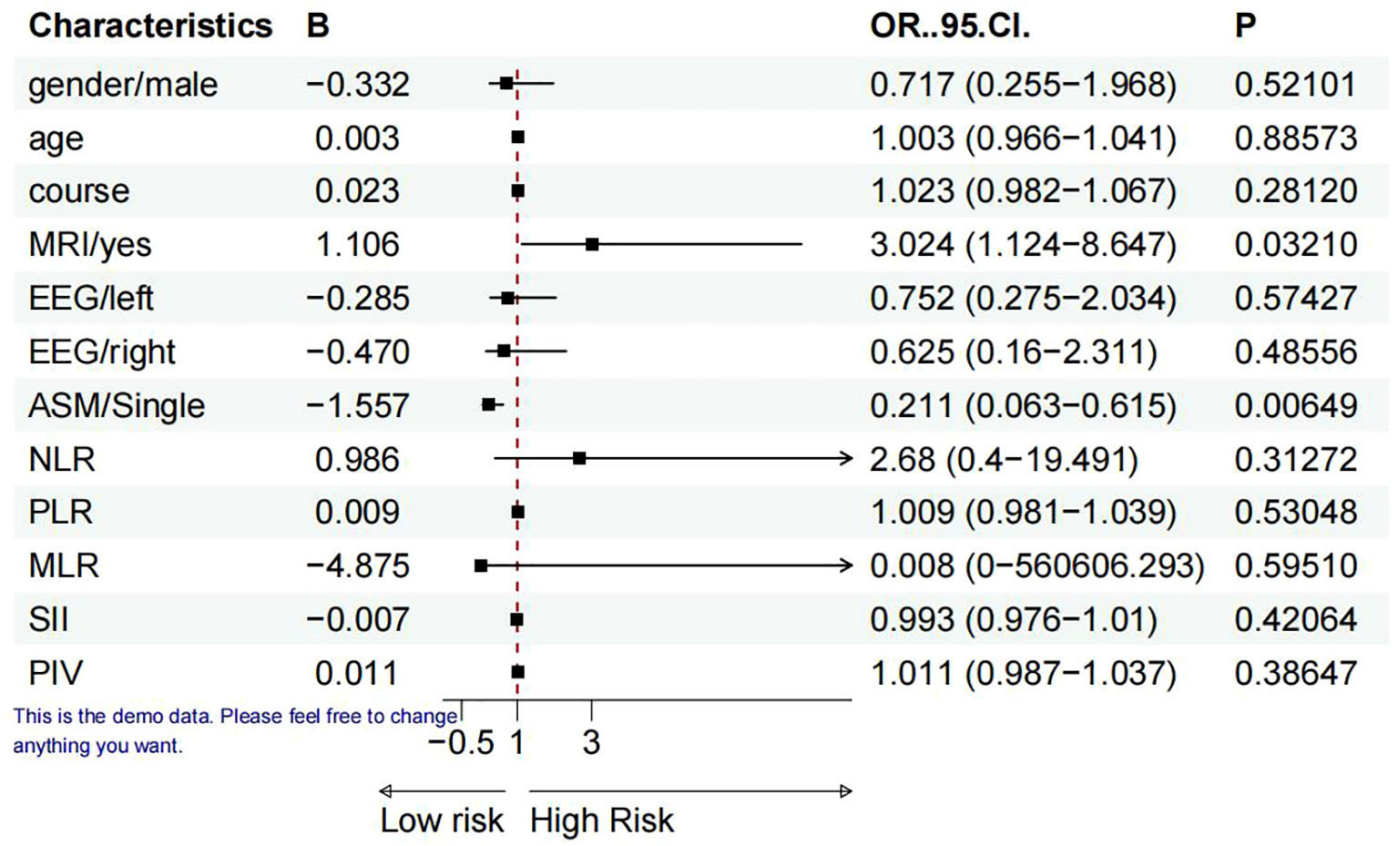
Predictors of TLE accompanied with personality alteration (forest plot). Variables utilized in binary logistic regression analysis: sex, age, disease progression, hippocampal MRI (hippocampal sclerosis or atrophy=yes), EEG (left and right), ASM, NLR, PLR, MLR, SII, and PIV.

